# Surge of Typhoid Intestinal Perforations as Possible Result of COVID-19–Associated Delays in Seeking Care, Madagascar

**DOI:** 10.3201/eid2712.210516

**Published:** 2021-12

**Authors:** Hyon Jin Jeon, Florian Marks, Jonathan Sugimoto, Justin Im, Sophie S.Y. Kang, Andrea Haselbeck, Raphaël Rakotozandrindrainy

**Affiliations:** University of Cambridge School of Clinical Medicine, Cambridge Biomedical Campus, Cambridge, UK (H.J. Jeon, F. Marks);; International Vaccine Institute, Seoul, South Korea (H.J. Jeon, F. Marks, J. Sugimoto, J. Im, S.Y. Kang, A. Haselbeck,);; University of Antananarivo, Antananarivo, Madagascar (F. Marks, R. Rakotozandrindrainy)

**Keywords:** typhoid, surveillance, *Salmonella enterica* serovar Typhi, Madagascar, intestinal perforations, COVID-19, coronavirus disease, SARS-CoV-2, severe acute respiratory syndrome coronavirus 2, viruses, respiratory infections, zoonoses

## Abstract

During the coronavirus disease pandemic, we observed a 6.4-fold increase in typhoid intestinal perforation incidence in Antananarivo, Madagascar. Thirteen perforations occurred within 6 months (February 2020–July 2020), compared with 13 perforations during the previous 41 months (August 2016–January 2020). The increase may be attributable to delayed healthcare seeking during the pandemic.

In an effort to understand the health impacts of endemic typhoid, the Severe Typhoid Fever Surveillance in Africa Program (SETA) detects and records cases of surgically confirmed intestinal perforations, a relatively rare but severe complication of *Salmonella enterica* serovar Typhi infection (*1*). Since the beginning of the coronavirus disease (COVID-19) global pandemic in early 2020, SETA surveillance has found an alarming increase in surgically confirmed intestinal perforations cases in Madagascar. This increase, which does not correlate with an increase in blood culture–confirmed typhoid cases found through SETA surveillance, may insinuate the serious effects on healthcare-seeking behavior and healthcare quality that the COVID-19 pandemic has had in the country.

Typhoid intestinal perforation is a severe complication of untreated or mismanaged infection that disproportionately affects low-income countries (*2*). Delay in diagnosis and proper antibiotic treatment of typhoid is frequently cited as a major factor contributing to typhoid intestinal perforation incidence and associated deaths (*3*–*6*). As such, increases in intestinal perforation cases may suggest deterioration in the quality of healthcare or changes in the healthcare-seeking behavior of the community.

SETA sentinel sites represent both primary and tertiary healthcare facilities, where all incoming patients are screened for febrile illness, clinically suspected typhoid, and gastrointestinal perforations. Once a patient is enrolled in the study, cultures of their blood, stool, and (in the case of surgery) tissue are used to detect *Salmonella *Typhi and other bacteremia. Over a 4-year period of SETA observation, we detected a marked increase in the rate of surgically confirmed typhoid intestinal perforations after the onset of the COVID-19 pandemic in early 2020 (Figure, panel A).

SETA Madagascar enrolled case-patients with suspected typhoid intestinal perforations in the town of Imerintsiatosika, 43 km from the capital city of Antananarivo, as well as case-patients from tertiary care facilities in Antananarivo (*1*) (Figure). Clinical and demographic data were systematically collected from enrolled participants at entry into the study. We observed participants daily until their hospital discharge. We detected a total of 26 intestinal perforation cases of any etiology during August 2016–September 2020. The mean age of the patients was 28.5 years (SD + 19.1 years); men and boys accounted for 69% of the total patients. Of the 26 patients with perforation, 9 died and 17 were discharged. The overall case-fatality rate was 50% among women and girls and 28% among men and boys. Of note, all 4 deaths among women and girls occurred during the prepandemic period, and all 5 deaths among men and boys occurred during the pandemic period. During August 1, 2016–January 31, 2020, the pre–COVID-19 pandemic period, 13 perforations (2.1/100,000 person-years of observation [PYO]) and 4 deaths (0.6/100,000 PYO) occurred. These incidence rates contrast with the remainder of 2020, the COVID-19 pandemic period, during which 13 perforations (13.2/100,000 PYO) and 5 deaths (5.1/100,000 PYO) occurred. This change represents a 6.4-fold (95% CI 3.0–13.7-fold) increase in the incidence of intestinal perforations during the COVID-19 pandemic period (p<0.05). Although we noted no statistically significant difference in mean age of onset for intestinal perforation patients detected before versus after the onset of the pandemic (30 vs. 27 years; p = 0.75 by t-test), intestinal perforation patients identified during the pandemic seem more likely to be middle-aged (20–50 years of age) (Figure, panel A).

**Figure:**
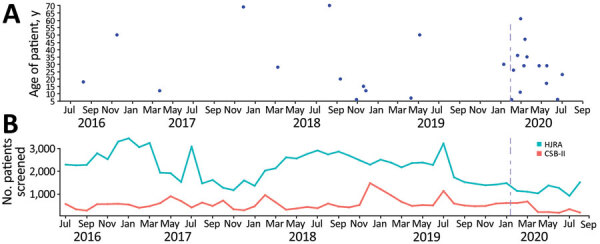
Typhoid intestinal perforation cases and number of patients screened in hospitals participating in the Severe Typhoid Fever Surveillance in Africa Program (SETA), Madagascar, July 2016–September 2020. A) Intestinal perforation cases recorded by SETA at 3 hospitals, by age of patient and date of hospitalization. B) Number of patients screened monthly by SETA at Hospital Joseph Ravoahangy Andrianavalona, the largest hospital in the capital city of Antananarivo, and its tertiary care center, and at the Centres Santé de Bases II, a primary care facility in the town of Imerintsiatosika in the rural region west of Antananarivo. Vertical purple lines indicate date first case of COVID-19 reported in Africa. CSB-II, Centres Santé de Bases II; HJRA, Hospital Joseph Ravoahangy Andrianavalona.

We suspect that the immediate increase in incident intestinal perforations observed since February 2020 may be an externality of delayed treatment for mild typhoid fever because of changes in healthcare-seeking behavior, healthcare quality, or both during the initial COVID-19 pandemic outbreak. However, COVID-19 investigations to date indicate that COVID-19 can affect various organs, including the gastrointestinal tract; hence, the possibility of SARS-CoV-2 having a direct effect on the risk for perforations cannot be ruled out at this stage and warrants further research.

As of Oct 19, 2021, a total of 42,898 COVID-19 cases and 958 COVID-19–related deaths had been reported in Madagascar (*7*). Well before the first 3 cases of COVID-19 were reported in-country on March 20, 2020, media coverage of the global pandemic was substantial (*8**,**9*). Like the rest of the international community, Madagascar watched with collective anxiety and apprehension as the novel coronavirus outbreak unfolded. Although the surge of reported perforation cases predates regional lockdowns, which were first imposed in July 2020 (*10*), we cannot rule out ad hoc closures of healthcare centers affecting the community’s ability to seek regular care in addition to the unknowns of potential social stigma that raises barriers to the already low levels of healthcare-seeking observed during nonpandemic conditions in the community (*11*,*12*).

Before future in-depth qualitative research can provide a comprehensive picture of healthcare in Madagascar during the COVID-19 pandemic, SETA screening records may provide the first hint at a disruption of individual healthcare-seeking behavior as shown by a reduction in the number of overall patient hospital visitations beginning in January 2020 (Figure, panel B). SETA records all patients who visit any of the sentinel health centers for any concern and screens those patients for study eligibility; consequently, SETA screening numbers can be understood as a proxy for hospital visitation numbers.

The COVID-19 pandemic is likely having broad impacts on other preventable diseases in an already struggling healthcare system; widespread availability of COVID-19 vaccines in Madagascar is expected only in 2023 (*13*). The observed increase in illness and deaths from treatable diseases and disruption of routine primary care should not be neglected (*14*,*15*). Although the SETA program has only investigated intestinal perforations in the capital city, delayed healthcare-seeking might be an even larger problem in more remote areas of the country. The public health community must remain vigilant about maintaining routine healthcare services and ensuring that healthcare facilities are safe and usable. In particular, public trust in the healthcare system amidst the pandemic is essential for encouraging persons with potentially life-threatening conditions to seek healthcare.
